# Beyond the Incision: Pediatric Postoperative Sepsis Risk Patterns and Related Adverse Events in U.S. Inpatient Care

**DOI:** 10.3390/healthcare13131595

**Published:** 2025-07-03

**Authors:** Michael Samawi, Gulzar H. Shah, Linda Kimsey

**Affiliations:** Department of Health Policy and Community Health, Jiann-Ping Hsu College of Public Health, Georgia Southern University, P.O. Box 8015, Statesboro, GA 30460, USA; msamawi1@georgiasouthern.edu (M.S.); lkimsey@georgiasouthern.edu (L.K.)

**Keywords:** postoperative sepsis, Pediatric Quality Indicators (PDIs), healthcare-associated infections, pediatric surgery, patient safety, health disparities, hospital characteristics, risk factors, HCUP Kids’ Inpatient Database (KID)

## Abstract

**Background**: Postoperative sepsis (POS) is a serious pediatric safety event tracked by the Agency for Healthcare Research and Quality’s Pediatric Quality Indicator 10 (PDI 10). **Purpose**: This study examined POS in United States inpatient care. **Methods**: Using the 2019 Kids’ Inpatient Database (KID), a nationally representative sample of U.S. pediatric discharges, we performed multivariable logistic regression to examine patient- and hospital-level predictors of POS. **Results**: Among approximately 5.24 million weighted discharges, 577,625 (12.65%) were flagged with POS. Key independent risk factors included undergoing major surgery, being treated in large urban teaching hospitals, and admission for surgical or injury-related care. Hospital characteristics such as Western region location, for-profit ownership, and large bed size were associated with increased POS odds, while rural and small hospitals showed protective effects. Race/ethnicity showed mixed findings; Native American and “Other” race patients had elevated POS risk, while Hispanic children had slightly reduced odds compared to White peers. **Conclusions:** Contrary to prior assumptions, neither age nor sex was a significant independent predictor. This study expands upon prior pediatric adverse event research by delineating the distinct risk profile of POS. The findings underscore the need for targeted infection control strategies in high-risk environments and support ongoing quality improvement efforts to reduce the surgical sepsis burden in children.

## 1. Introduction

Postoperative sepsis (POS) in pediatric patients is a severe complication defined as a systemic inflammatory response syndrome due to infection occurring after surgery. It aligns with the Surviving Sepsis Campaign definition of life-threatening organ dysfunction from a dysregulated host response to infection [1]. Pediatric POS may arise from surgical site infections, central line infections, urinary or respiratory infections after an operation, and it often requires intensive care interventions. Early identification and treatment are critical to prevent progression to septic shock or multiorgan failure [1].

Prior research has highlighted that postoperative sepsis in children has distinct characteristics compared to sepsis in nonsurgical patients. In a cohort of pediatric severe sepsis cases, those following surgery tended to be younger and have more gastrointestinal or cardiovascular comorbidities than medical sepsis cases [2]. Postsurgical sepsis was more often associated with abdominal infection sources and Gram-negative organisms [2]. Notably, although mortality was similar between the postsurgical and medical sepsis groups, the risk factors for death differed: postsurgical sepsis outcomes were worse in children with cardiovascular or respiratory comorbidities and in older children, whereas medical sepsis mortality was linked to hospital-acquired infections, higher illness severity upon admission, oncologic comorbidities, and resource-limited settings [2]. Postsurgical sepsis cases also incurred extended hospital stays and greater resource utilization [2]. These findings underscore that sepsis after surgery constitutes a unique entity with specific risk profiles, meriting focused study as a standalone adverse event.

Targeted infection prevention strategies have shown promise in reducing postoperative and hospital-acquired infections in pediatric surgical populations. For example, the implementation of standardized central line-associated bloodstream infection (CLABSI) prevention bundles in a pediatric cardiac surgery unit significantly reduced the bloodstream infection rate [3]. However, the impacts on surgical site infections (SSIs) and ventilator-associated pneumonia were less pronounced [3]. Identified risk factors for central postoperative infections in that setting included younger age at surgery, prolonged ventilation, and the use of a central line [3]. These results underscore the importance of evidence-based bundles and vigilant monitoring to improve pediatric surgical outcomes.

The Agency for Healthcare Research and Quality (AHRQ) has developed Pediatric Quality Indicators (PDIs) to systematically track adverse events, including infections and respiratory complications [4]. Three of the most prevalent PDIs are neonatal bloodstream infection (NBSI, AHRQ NQI 03), pediatric postoperative respiratory failure (PORF, PDI 09), and pediatric postoperative sepsis (POS, PDI 10) [4]. Recent analyses of national data have examined the first two of these indicators [5,6,7]. Neonatal bloodstream infection (NBSI) primarily affects newborns (often related to central lines and neonatal intensive care) and was found to be the single most frequent pediatric safety event in 2019 (rate of ~331 per 10,000 discharges) [5,7]. Postoperative respiratory failure (PORF) is a serious complication of pediatric surgery and had the second-highest incidence (~268 per 10,000 discharges in 2019) [5,6]. Meanwhile, POS has been observed to be at a rate of ~114.9 cases per 10,000 discharges [5]. It is worthwhile to note that these rates represent population-level, unadjusted numerators divided by the total pediatric discharge denominator, which helps to contextualize the burden of each indicator [5]. Rates represent the number of POS cases per 10,000 total pediatric discharges, consistent with the PDI reporting methodology. Our previous studies focusing on NBSI and PORF in the 2019 inpatient dataset identified several key risk factors: large urban teaching hospitals and for-profit hospitals had higher odds of these adverse events, whereas small, rural hospitals had lower odds [6,7]. We also observed differences in patient-level factors—Black children experienced higher odds of NBSI compared to White ones, while PORF risk was elevated in Hispanic children in adjusted analyses [6,7]. These prior findings suggest that infection-related complications (like NBSI and POS) and respiratory complications (PORF) may share some systemic risk factors (such as hospital environment and patient comorbidity burden) but also differ in their demographic and clinical associations.

Emerging evidence suggests significant disparities in pediatric postoperative outcomes across different populations. A recent national study by Parikh et al. (2024) reported that Black and Hispanic children have significantly higher odds of developing safety events, including POS, compared to White children when adjusting for other factors [5]. In that analysis of the 2019 Kids’ Inpatient Database, Black patients had (55%) higher odds of POS than Whites, and children on Medicaid had (45%) higher odds of POS than those with private insurance [5]. These disparities persisted even when stratifying by payer, indicating that racial/ethnic minority children face elevated risks that are not explained solely by insurance status [5]. Such findings underscore the urgent need to investigate POS with an equity lens and to identify modifiable factors in care delivery that contribute to these gaps.

The risk of POS in pediatric patients is a function of a complex interaction of clinical factors (e.g., type of surgery, comorbidities), hospital structural characteristics (e.g., size, region, and ownership), and patient sociodemographic attributes (e.g., race/ethnicity, insurance status) [8]. This complex set of factors may influence both exposure to sepsis-related risks and the reactivity of the healthcare system, suggesting that discrepancies in POS outcomes may indicate systemic inequities rather than only isolated clinical differences.

In this study, we build upon the prior analyses of PORF and NBSI by examining POS (PDI 10) as an independent outcome in pediatric inpatient care. Our objectives are to determine the incidence and characteristics of POS in a nationally representative pediatric cohort and to identify patient and hospital factors associated with its occurrence. We also sought to compare the risk factor profile of POS to that of PORF and NBSI, to discern shared versus divergent patterns in infection-related adverse events. By answering these questions, we aim to inform targeted strategies for improving surgical safety in children and reducing the burden of postoperative complications (POS) in pediatric hospitals.

## 2. Materials and Methods

### 2.1. Study Design and Data Source

We conducted a retrospective cohort study using the 2019 Healthcare Cost and Utilization Project (HCUP) Kids’ Inpatient Database (KID), which is the largest all-payer pediatric inpatient database in the United States [9]. The KID contains a nationally representative 20% stratified sample of pediatric discharges (patients ≤ 20 years old) from 4000+ U.S. community hospitals, including general, specialty, and children’s hospitals (excluding federal or long-term facilities) [9]. The 2019 KID includes over 3 million unweighted pediatric discharges, which represent approximately 6–7 million weighted hospitalizations nationwide after applying survey weights [9]. This database captures detailed patient and hospital information for each admission, including up to 40 diagnoses and 25 procedures coded with ICD-10-CM/PCS, as well as hospital characteristics [9]. The study was granted exempt status by the Georgia Southern University Institutional Review Board (Protocol H23359) because the KID is a de-identified public dataset with no direct patient identifiers.

### 2.2. Outcome Variable

The outcome analyzed in this research is postoperative sepsis (POS), identified according to AHRQ PDI 10 specifications: cases of sepsis (secondary diagnosis of an ICD-10 sepsis code) in surgical patients aged 17 or younger, excluding patients with a principal diagnosis of sepsis or an infection present upon admission, neonates, and obstetric admissions [4]. In practice, PDI 10 captures children who developed sepsis during hospitalization after a surgical procedure [4]. We used the AHRQ Pediatric Quality Indicator software (version 2020) implemented in SAS 9.4 to flag cases of PDI 10 based on the discharge diagnoses and procedures. The POS outcome was treated as a binary dependent variable (1 = POS occurred, 0 = no POS occurred).

### 2.3. Independent Variables

Based on prior research and data availability, we examined a range of hospital-level and patient-level characteristics as independent variables [6,7]. Hospital characteristics extracted from the KID hospital file included (1) bed size (small, medium, and large, defined by HCUP cut-offs based on hospital volume and region), (2) location/teaching status (rural, urban non-teaching, and urban teaching hospital), (3) region (Northeast, Midwest, South, and West), and (4) ownership type (public/government, private not-for-profit, and private for-profit investor-owned). Patient characteristics included age in years (treated as a continuous variable in regression), sex (male or female), race/ethnicity (White, Black, Hispanic, Asian/Pacific Islander, Native American, and Other/Unknown), primary service line of the admission (Maternal/Neonatal, Medical, Surgical, Mental Health/Substance Use, or Injury—as classified in the KID based on diagnosis-related groups), primary payer (Medicaid, private insurance, self-pay, Medicare, other, or no charge), and whether a major operating room procedure was performed during the admission (yes/no). Notably, the Maternal/Neonatal service line primarily represents birth hospitalizations and neonatal care; such admissions were included in the dataset for completeness, but neonates are not at risk of PDI 10 by definition (since POS excludes neonatal cases) [4]. To capture surgical exposure beyond the PDI 10 criteria, we used the HCUP data element PCLASS_ORPROC, which flags discharge records containing at least one major diagnostic or therapeutic operating room procedure based on ICD-10-PCS codes, as defined by the DRG grouper. This binary indicator served as a general proxy for surgical intervention during hospitalization. Full details of the classification system are provided in Appendix A.

### 2.4. Analysis

We first conducted descriptive analyses to characterize the distribution of patient demographics, hospital types, and the occurrence of POS in the 2019 sample. The incidence of POS was calculated as the proportion of discharges meeting the PDI 10 criteria among all pediatric discharges. Bivariate analyses (chi-square tests and *t*-tests) were used to compare characteristics between cases with POS and those without. We then built a multivariable logistic regression model to identify independent associations between the covariates and the odds of POS. All candidate variables listed above were entered into the model. Adjusted odds ratios (AORs) with 95% confidence intervals were estimated for each predictor. The regression was weighted using the HCUP-provided discharge weights to produce national estimates, and standard errors were adjusted for the complex survey design of the KID. A two-sided *p*-value < 0.05 was considered statistically significant. Results are reported as odds ratios with corresponding 95% confidence intervals. Analyses were performed using SAS version 9.4 [10].

## 3. Results

Patient and hospital characteristics: In 2019, the KID captured approximately 5.24 million weighted pediatric discharges nationwide. Among these, we identified POS (PDI 10) taken at the numerator level, in 577,625 discharges, which corresponds to roughly 12.65% of all pediatric discharges taken at the numerator level.

Table 1 summarizes the cohort characteristics. Briefly, the majority of pediatric discharges occurred in large hospitals (about 61%) and urban teaching hospitals (82%). Regionally, the South accounted for the largest share of pediatric stays (38%), followed by the West (22%), the Midwest (23%), and the Northeast (17%). Most hospitals were private not-for-profit (77%), with 12% being public and 11% being investor-owned facilities. Just over half of the patients were female (51.4%). The racial/ethnic composition of hospitalized children was 45.6% White, 19.7% Hispanic, 16.6% Black, 4.0% Asian/Pacific Islander, 0.9% Native American, and about 6.1% identified as “Other” or multiracial. Reflecting the inclusion of newborn care in the dataset, 55.6% of discharges were classified under the Maternal/Neonatal service line (which includes routine newborn births and neonatal ICU cases). In comparison, 27.4% were medical (non-surgical pediatric illnesses), 7.1% were surgical (non-neonatal surgeries), 6.8% were mental health-related, and 3.2% were injury-related. In terms of payer mix, approximately 50.7% of pediatric stays were covered by Medicaid, 41.1% by private insurance, 4.3% were self-pay/uninsured, 3.3% by other payers, and a small fraction by Medicare (0.3%—e.g., children with certain disabilities) or no-charge/charity care (0.1%). Not every hospitalization involved a surgical procedure: overall, 12.0% of pediatric discharges had at least one major operating room procedure recorded, while 88.0% had no major surgery. As expected, the occurrence of POS was concentrated within that surgical subset.

### Multivariable Analysis of POS

Table 2 presents the adjusted odds ratios from the logistic regression for PDI 10 (POS). Our final multivariate model had a Nagelkerke R^2^ of 0.0673, which is typical for a study that uses big administrative datasets and lacks detailed clinical variables. While the model’s c-statistic of 0.683 suggests modest discrimination, this level of performance is consistent with models derived from secondary administrative data, which lack certain clinical variables such as laboratory results, infection markers, or severity scores. Several patient and hospital factors showed significant independent associations with the likelihood of POS (*p* < 0.01 for most predictors). Notably, patient age and sex were not significant predictors of postoperative sepsis after adjusting for other factors (*p* = 0.5780 and *p* = 0.2823, respectively), indicating that older children did not have a statistically different risk of POS compared to younger ones, and females had similar odds as males when other variables were held constant.

Regarding patient race/ethnicity, most groups did not differ substantially from the reference category (White) in the adjusted model, with two exceptions. *Hispanic* children showed a slightly *lower* odds of experiencing POS compared to White children (AOR = 0.969, 95% CI: 0.961–0.977, *p* < 0.0001). In contrast, *Native American* children had significantly higher odds of POS (AOR = 1.084, 95% CI: 1.054–1.114, *p* < 0.0001). Patients classified as “Other” race (including multiracial or not elsewhere specified) also had higher odds (AOR = 1.059, 95% CI: 1.046–1.072, *p* < 0.0001). The odds for *Black* patients and *Asian/Pacific Islander* patients were not significantly different from Whites after adjustment (their AORs were near 1.0 with *p* > 0.05). These results suggest subtle but significant racial differences, particularly a protective effect in the Hispanic population and elevated risk in Native Americans for POS, when controlling for other factors.

All categories of the admission service line were strongly associated with POS risk (*p* < 0.0001). Using medical admissions as the reference, children admitted for *Surgical* reasons had the highest likelihood of POS (AOR = 1.314, 95% CI: 1.291–1.337), followed closely by those in the *Injury* service line (AOR = 1.261, 95% CI: 1.241–1.281). This indicates that patients hospitalized for surgical procedures or trauma-related care had about 26–31% higher odds of developing sepsis postoperatively compared to those admitted for general medical conditions, all else being equal. In contrast, patients in the *Maternal/Neonatal* service line had a substantially lower risk (AOR = 0.687, 95% CI: 0.682–0.692) compared to medical patients. Similarly, those in *Mental Health/Substance Use* admissions had lower odds (AOR = 0.787, 95% CI: 0.776–0.798) of POS. These findings reflect the fact that neonatal births and psychiatric admissions involve far fewer invasive procedures and less risk of nosocomial infection than surgical or injury-related admissions.

Primary payer showed nuanced effects on POS odds. Compared to the reference group of self-pay patients (uninsured), children with *Medicaid* as the payer had a slightly reduced adjusted risk of POS (AOR = 0.981, 95% CI: 0.967–0.995, *p* = 0.0073), suggesting no increase in POS despite Medicaid patients often presenting with higher baseline illness severity. In contrast, children with *private insurance* had a modest but statistically significant increase in odds of POS (AOR = 1.043, 95% CI: 1.028–1.059, *p* < 0.0001) relative to self-pay. Two smaller groups—“*No charge*” patients (e.g., charity care) and “*Other*” *payers* (e.g., *miscellaneous programs*)—also showed higher odds of POS. Notably, “No charge” encounters had an AOR of about 1.36 (95% CI: 1.265–1.454), the most significant increase among payer groups, though this category represented a minimal number of discharges. Patients under *Medicare* (primarily children with certain chronic conditions) did not have a significant difference in POS risk compared to self-pay patients in this model.

The presence of a major operative procedure during hospitalization was a strong predictor of postoperative sepsis. Patients who underwent at least one major surgery had an adjusted odds of POS about 1.18 times that of patients without a major operating room procedure (AOR = 1.177, 95% CI 1.160–1.194, *p* < 0.0001). In other words, the odds of developing sepsis were approximately 18% higher if a major surgery was performed, controlling for all other factors. This underlines that the operative nature of an admission is inherently a risk factor for severe infection complications.

Several hospital-level characteristics were significantly associated with POS in adjusted analyses. The *hospital bed size* analysis showed that smaller hospitals have markedly lower odds of pediatric POS compared to large hospitals. Being hospitalized in a *small pediatric hospital* was associated with roughly a 28% decreased odds of POS (AOR = 0.718, 95% CI: 0.712–0.723), and in a *medium-sized hospital* with about a 21% decrease (AOR = 0.786, 95% CI: 0.780–0.792), both relative to large hospitals (*p* < 0.0001). There was a clear gradient whereby large tertiary hospitals carried the highest risk for POS events.

*Hospital location and teaching status* had an even more pronounced effect. Children admitted to *rural hospitals* experienced substantially lower odds of POS (AOR = 0.364, 95% CI: 0.360–0.368) compared to those in *urban teaching hospitals* (*p* < 0.0001). Even *urban non-teaching hospitals* had significantly reduced odds (AOR = 0.520, 95% CI: 0.515–0.525) compared to urban teaching centers. These ratios indicate that the risk of POS in a rural hospital was approximately one-third that in an urban academic center, and in an urban community hospital it was about one-half, after adjusting for patient case mix and other factors.

There were also notable differences by *geographic region*. Taking the Western U.S. as the reference region (which had the highest unadjusted POS rates), the *Midwest* region had about 22% lower odds of POS (AOR = 0.775, 95% CI: 0.768–0.782) and the *South* had about 20% lower odds (AOR = 0.802, 95% CI: 0.795–0.808) compared to the West (*p* < 0.0001 for both). The *Northeast* region showed a smaller but still significant reduction (AOR = 0.824, 95% CI: 0.817–0.832). These regional effects suggest that pediatric patients in the West experienced the highest risk of POS. In contrast, those in other regions—particularly the Midwest—had comparatively lower risk, even after accounting for hospital and patient characteristics.

Finally, *hospital ownership* was associated with slight differences in POS outcomes. Relative to *private for-profit (investor-owned) hospitals*, being treated in a *private not-for-profit* hospital was associated with approximately a 9% lower odds of POS (AOR = 0.910, 95% CI: 0.902–0.919, *p* < 0.0001). *Public (government-owned) hospitals* also showed a small protective effect (OR = 0.979, 95% CI: 0.968–0.991, *p* < 0.001) compared to investor-owned facilities. Thus, for-profit hospitals had the highest adjusted rates of pediatric POS, whereas not-for-profit and public institutions had slightly better outcomes in this regard.

**Figure 1:**
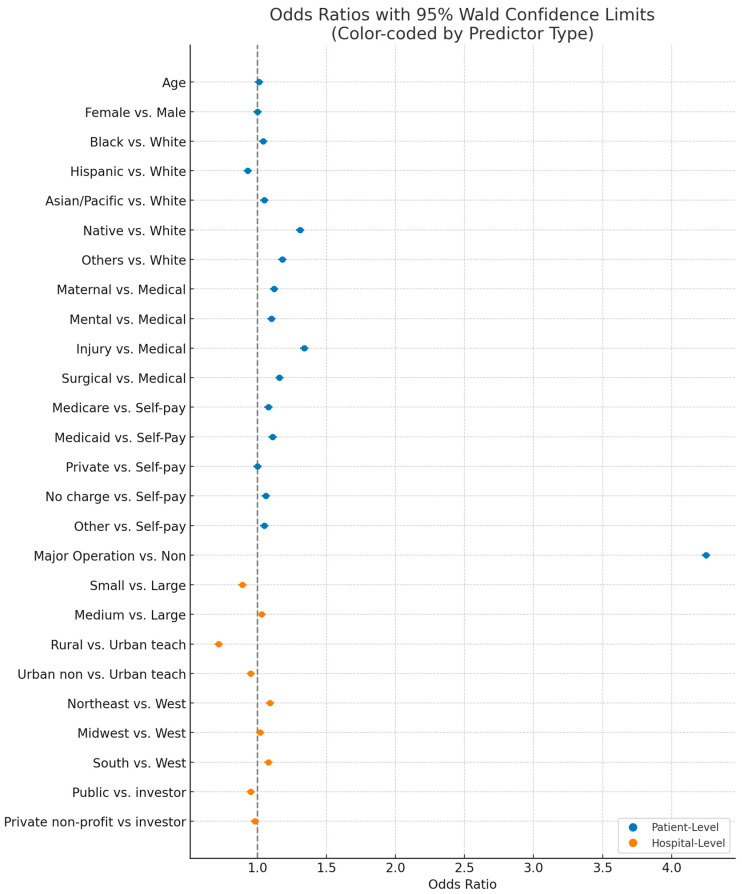
Independent-variable odds ratio with 95% Wald confidence limits associated with POS (multivariate).

Figure 1 shows adjusted odds ratios with 95% Wald confidence limits for factors associated with pediatric POS (PDI 10) in 2019. Blue dots represent the estimated odds ratio (on a log scale) for patient characteristics, while orange dots indicate those of the hospital-level characteristics. The surrounding bars indicate the 95% confidence interval. The vertical line at 1.0 indicates no difference in odds. Values to the right of the line (OR > 1) denote higher odds of POS for that category versus the reference, while values to the left (OR < 1) denote lower odds.

The multivariate findings reinforce the results of the unadjusted analysis while highlighting which factors retain independent significance. In the next section, we compare these results with those of prior studies and discuss their implications for pediatric patient safety.

## 4. Discussion

In this nationally representative analysis of pediatric inpatient data, we identified multiple factors associated with POS, shedding light on which children and hospitals are at highest risk for this serious adverse event. The percentage found for the occurrence of POS was lower than that of NBSI (the most frequent pediatric adverse event, at 21.39% of neonate patients) yet higher than PORF (at 7.69% of pediatric discharges) in the same dataset [6,7]. This, nonetheless, represents a substantial number of affected children nationwide. In absolute terms, over half a million pediatric patients developed sepsis after a surgical procedure in 2019.

A key finding regarding POS is that contextual and system factors—such as hospital type, size, and region, as well as the nature of the admission (surgical vs. medical)—emerged as dominant predictors of this adverse event. In contrast, individual patient factors, such as age and sex, were not independently predictive after adjustment. This aligns with our earlier studies on pediatric PORF and NBSI, which likewise found that the hospital environment and admission characteristics significantly influenced adverse event rates [6,7]. The present study’s focus on PDI 10 allows for direct comparisons with those prior results, revealing both shared patterns and significant divergences in risk profiles across different pediatric safety indicators.

Consistent with the PORF analysis [6], we observed that being treated at a large, urban teaching hospital was associated with higher odds of POS. These institutions often care for the most complex cases—children with severe comorbidities, undergoing high-risk surgeries, which likely explains the elevated rates of complications. Smaller and non-teaching hospitals had substantially lower POS rates, mirroring patterns seen for PORF and NBSI [6,7]. It is essential to note that this does not necessarily indicate superior quality of care at smaller hospitals; rather, it reflects case mix differences and the concentration of tertiary as well as quaternary care in major centers. The sickest children (e.g., those with congenital heart disease, oncology patients, and trauma cases) are often transferred to large academic hospitals where they undergo interventions that carry greater infection risk. Our findings reinforce that pediatric patient safety metrics, such as POS, are heavily influenced by where care is delivered and to whom. Quality improvement efforts may thus need to be especially intensive in large referral centers, focusing on the complex care processes that such high-risk patients require.

Interestingly, patient sex and age were not significant predictors of POS in our adjusted model, indicating that boys and girls, as well as children of different ages, had comparable risks when other factors were controlled. This finding aligns with our PORF study, where sex differences disappeared after adjustment [6], and with our NBSI study, which found only a minimal difference (a slightly higher unadjusted NBSI rate in male infants) [7]. The lack of a strong sex effect for operative complications suggests that factors other than inherent physiological sex differences are far more critical in determining outcomes. This result runs somewhat contrary to earlier reports that suggested female gender independently predicted lower postoperative morbidity, but more-extended hospital stays and higher total charges [11]. While prior studies have shown variable gender effects across pediatric conditions (e.g., higher male mortality in preterm infants, higher resource use in male patients with sinusitis), these studies together emphasize the need to include gender in pediatric surgical risk models dependent upon types of surgeries performed [11]. Nonetheless, those studies involved broader contexts and did not specifically focus on POS. Our analysis implies that once we account for the clinical scenario and hospital setting, sex alone contributes little to predicting POS. In practical terms, this means quality improvement measures should be applied equitably to boys and girls, with lower need for sex-specific modifications in sepsis prevention protocols at the bedside.

Patient age similarly did not affect POS risk after adjustment, which is notable given the broad age span of pediatric patients (from neonates through adolescents). In our previous work, age was a significant factor for PORF, with infants and younger children at a higher risk of respiratory failure after surgery [6]. For POS, however, age did not show a significant independent effect. This could be because the PDI 10 indicator inherently excludes neonates and focuses on older children, among whom age-related physiological differences (e.g., immune maturity) may be less pronounced than the extreme contrast between neonates and others in NBSI. It is worth recalling that the separate NQI 03 indicator effectively captures neonatal sepsis, and our NBSI study dealt exclusively with the neonatal population [7]. When considering POS (which, by definition, excludes neonates), the age range is restricted to infants over 1 month old up to adolescents. Within this group, we found no strong age-related trend in sepsis risk. This suggests that all pediatric age subgroups undergoing surgery are vulnerable to POS, and vigilance should not be relaxed based on age alone. Even teenagers, despite having more mature immune systems, did not have significantly lower adjusted risk than younger children in our study.

Racial and ethnic disparities in pediatric safety events have been a focus of recent research, and our findings provide a nuanced picture for POS. In the bivariate context, Black and Hispanic children had higher raw rates of POS than White children, consistent with the national disparities reported by Parikh et al. [5]. However, in our multivariable model (accounting for hospital and clinical factors), Black patients’ odds of POS were not significantly different from Whites’, and Hispanic patients surprisingly showed slightly *lower* odds than Whites. This contrasts with Parikh’s study, which found a 55% higher odds of POS in Black children and a modest increase in Hispanic children’s risk in an adjusted model [5]. One possible explanation for this difference is that our model included a more extensive set of hospital characteristics and admission factors that may mediate the relationship between race and outcomes. For example, Black children are over-represented in specific regions or hospital types that have higher complication rates; once we adjust for those variables, the direct effect of race might diminish. Indeed, in our earlier NBSI analysis, being Black was a significant risk factor for neonatal sepsis (AOR ~1.3 vs. whites) [7], yet for the operative indicators (PORF and POS) we did not see a significant Black–White gap after full adjustment. On the other hand, the Native American pediatric population consistently showed elevated risks in our analyses; here, we found Native American children had ~8% higher odds of POS compared to Whites, as we also noted for PORF [6]. This suggests that there may be unmeasured factors (such as a higher burden of comorbid conditions or access issues) affecting Native American children undergoing surgery. The “*Other*” race category also had increased POS risk, which could indicate that multiracial or not-categorized children (or possibly those for whom race data were missing) might be capturing certain vulnerable groups.

It is essential to interpret these racial and ethnic findings in context. The Hispanic paradox in our results—where Hispanic children had a slightly lower adjusted risk of POS—might reflect demographic and health-system factors; for instance, Hispanic families may disproportionately use certain hospitals or could have differing baseline health statuses that are not fully captured in administrative data. Parikh et al. reported that Hispanic children had higher odds of the other examined postoperative adverse event, PORF (respiratory failure) [5], so it is possible that, for different outcomes, the disparity patterns vary. Further research using datasets with richer clinical and socioeconomic information, or applying alternative statistical modeling approaches, is warranted to better understand these observed differences. In our study, Asian/Pacific Islander children did not have a significant difference in POS after adjustment, even though prior work noted higher rates of some PDIs in this group [5]. Altogether, our results underscore that racial disparities in pediatric POS exist, but they are complex and intertwined with other factors like hospital context and case mix. The persistent higher risk for Native American patients, as well as the large unadjusted disparities for Black and Hispanic patients, indicate a need for targeted quality improvement and equity-focused interventions. Ensuring that all children receive optimal surgical care and postoperative monitoring, regardless of race/ethnicity, is a key public health priority.

The type of clinical service line was one of the strongest predictors of POS in our analysis. Children admitted for surgical or injury-related care had significantly higher odds of POS. In contrast, those in Maternal/Neonatal or Mental Health service lines had much lower odds (by 20–30% or more in adjusted terms). This makes intuitive sense: *surgical* and *trauma* patients undergo invasive procedures and often require intensive postoperative support (ventilation, central lines, etc.), which increases infection risk. Our findings are consistent with earlier observations that high-complexity surgeries in children carry substantial risks of complications [6,7]. For instance, in smaller-scale pediatric studies, primary operations (like cardiac or neurosurgery) have been linked to higher infection rates and respiratory complications postoperatively. The *Maternal*/*Neonatal* category in our dataset primarily comprises healthy newborn stays and neonatal intensive care. Healthy newborns rarely undergo surgery, and neonatal sepsis (although common in NICUs) is captured under NBSI rather than POS. Thus, it is logical that this group showed low POS rates. *Mental Health* admissions also have a very low risk, since they typically do not involve surgical procedures or indwelling devices. One interesting finding was that the *Injury* service line had elevated POS risk (AOR ~1.26), suggesting trauma cases are particularly prone to postoperative infections, likely due to emergency surgeries, open wounds, or the physiology of injury and shock. Injury-related admissions did not show a significant increase in NBSI in our previous work (as neonates are seldom injury patients) [7], but they did have higher PORF odds in at least one model [6]. Taken together, these results highlight that the complexity and urgency of the clinical scenario (planned surgery vs. urgent trauma vs. routine medical care) influences the likelihood of severe complications like sepsis. This highlights the need for tailored preventive measures, such as enhanced infection surveillance and prophylaxis in trauma surgery cases, as well as specialized care pathways for surgical patients to mitigate sepsis risk.

Primary payer status yielded some unexpected findings. We initially anticipated that children with public insurance (Medicaid) might have higher adverse event rates, given that Medicaid status is often associated with lower socioeconomic status and has been linked to higher pediatric adverse event incidence in past research [12]. Indeed, in our NBSI study, Medicaid-covered neonates had higher odds of bloodstream infection compared to privately insured ones [7], and Parikh et al. also found that Medicaid was associated with greater odds of several PDIs (including a 45% higher odds of POS) [5]. However, in the current POS analysis, *Medicaid patients did not have increased adjusted risk*; in fact, they had slightly lower odds of POS than self-pay patients. Meanwhile, *privately insured patients showed a small but significant increase in odds of POS* relative to self-pay patients. One possible interpretation is that the reference group—self-pay—may represent a relatively healthier or differently managed population (for example, some elective surgery patients who self-pay might be international medical tourists or self-pay by choice, rather than indigent patients). Alternatively, this finding could be an artifact of coding or residual confounding; privately insured children might cluster at specific hospitals or have procedures (like elective surgeries) that still carry sepsis risk. It is also worth noting that the absolute differences were small (AORs of ~0.98 for Medicaid and 1.04 for private), so these may not translate into significant clinical differences. Nonetheless, the result that Medicaid was not a risk factor for POS is noteworthy and somewhat reassuring. It suggests that, at least for POS, once hospitalized and undergoing surgery, children on Medicaid received care with outcomes comparable to privately insured peers in 2019. This could reflect the widespread implementation of standardized surgical safety and infection control practices across hospitals, irrespective of payer mix. Still, given previous findings of Medicaid-related disparities, further investigation is needed. Medicaid status may affect other outcomes (such as NBSI or overall adverse events) more than it does POS specifically. The elevated odds for the “No charge” and “Other” payer groups in our study hint that system factors (such as hospital financial resources or care coordination for uninsured patients) might play a role in sepsis outcomes. Future work may explore whether hospitals with high uninsured burdens struggle more with complications or if there are differences in documentation.

Perhaps the most clinically actionable finding is the impact of major surgery on sepsis risk. We found that children who had a major operating room procedure were significantly more likely to develop sepsis, which is intuitive and aligns with abundant literature on postsurgical infection risks. This association held not only for POS but was also a common thread in our analyses of PORF and NBSI [7]. Essentially, undergoing invasive surgery greatly increases a child’s vulnerability to adverse events, whether respiratory failure, sepsis, or other complications. This underscores two key points: First, the *prevention and early management of sepsis should be core components of perioperative care* in pediatrics. Bundled interventions—such as strict sterile technique, timely antibiotics for surgical prophylaxis, vigilant postoperative monitoring for signs of infection, and the early activation of sepsis protocols—are critical for surgical patients. Second, clinicians should exercise caution when performing multiple or non-urgent additional procedures in an already-hospitalized child, as each additional major surgery confers an incremental risk. Our data emphasize the importance of making informed decisions regarding reoperation or extensive surgical interventions, especially in patients with a complicated postoperative course. When repeat surgery is necessary, it may be prudent to optimize the patient’s condition as much as possible and to prepare heightened surveillance for sepsis in the postoperative period.

The parallel examination of NBSI and POS also highlights the pervasive issue of healthcare-associated infections in children. Two out of the three top pediatric adverse events in 2019 (NBSI and POS) are sepsis-related, indicating that infection control remains a paramount challenge. While our study did not directly evaluate specific infection prevention practices, the results support ongoing efforts to implement sepsis reduction initiatives in pediatric hospitals. For neonates, this might include rigorous CLABSI prevention bundles, since many neonatal BSIs are central-line-related [3]. For postoperative patients, “sepsis bundles”—multifaceted protocols for the early recognition and management of sepsis—can be lifesaving. Recent quality improvement programs in adult and pediatric care have shown that bundle adherence (e.g., timely antibiotics, fluid resuscitation, source control for infection) improves sepsis outcomes. Our findings encourage pediatric healthcare providers to adopt a *holistic, proactive approach to sepsis prevention* that spans the continuum of care: from preoperative optimization (treating any existing infections before surgery), to intraoperative sterility and appropriate prophylaxis, to postoperative monitoring and early intervention at the slightest hint of sepsis. The concept of “*zero tolerance*” for healthcare-associated infections, championed in adult intensive care unit (ICU) settings, should also be applied in pediatric surgical units [13]. Every central line, surgical incision, and urinary catheter in a child is a potential entry point for infection and deserves meticulous attention [13].

Beyond clinical factors, our study reinforces the influence of hospital structural characteristics on pediatric outcomes. Large bed size, urban teaching status, and location in the Western US were each associated with higher odds of POS. The Western region finding aligns with our PORF analysis [6] and could reflect regional practice variations or population differences (for example, the West may have larger referral centers and perhaps different case mixes). Interestingly, our NBSI study found that the South had higher odds of neonatal sepsis [7], whereas for POS, the South had lower odds compared to the West. This suggests that regional risk may differ by outcome type, possibly related to regional differences in neonatal intensive care versus surgical care. It would not be surprising if certain regions have more robust infection prevention programs or if there are differences in coding practices. The consistently lower risk observed in the Midwest and Northeast for POS might indicate best practices or structural advantages in those regions that are worth emulating elsewhere.

The ownership effect, with private for-profit hospitals faring worse in POS, is also noteworthy. For-profit hospitals may face resource constraints or different operational priorities that affect nurse staffing, infection control investments, or case mix. Public and not-for-profit children’s hospitals, often with academic affiliations and potentially more access to state or federal support or philanthropic funding, may invest more in safety infrastructure. We cannot determine causality here, but our findings align with some prior research suggesting not-for-profit hospitals have better quality outcomes on average than for-profit hospitals in pediatrics [12]. Policymakers and hospital administrators should be aware that organizational factors and financial models can have a ripple effect on patient safety. Transparency in quality metrics and the sharing of best practices across hospital types can help raise the bar, especially for institutions that may be lagging behind.

### 4.1. Public Health Implications

The insights from this study should be leveraged to improve pediatric surgical safety on several fronts. First, targeted interventions are needed for high-risk groups and settings. Our data highlight specific combinations—e.g., a child with complex comorbidities undergoing major surgery at a busy urban teaching hospital—that are particularly prone to sepsis. Hospitals should stratify risk (using criteria similar to our model) and allocate extra preventive resources accordingly. For instance, enhanced sepsis screening protocols or prophylactic measures could be targeted at intensive care units and surgical wards in large teaching hospitals, where most POS cases occur. At a system level, structural interventions aimed at reducing disparities are crucial [14]. This could mean investing in healthcare access and preventive care for racial/ethnic minority children and those from low socioeconomic backgrounds, to optimize their health status before surgery and ensure the timely treatment of complications. Community-level interventions—for example, improving transportation and access to specialty care for rural or underserved populations—may help mitigate some of the upstream risk factors that contribute to poorer hospital outcomes [15].

Second, continuous education and training for pediatric healthcare providers is paramount. Frontline staff should be well versed in pediatric early warning signs, sepsis protocols, and infection prevention techniques. Ongoing training initiatives and competency assessments (for example, simulation exercises for recognizing postoperative sepsis in children) can maintain high awareness [16]. Given the fast turnover of evidence, hospital leadership should cultivate a culture of learning where new guidelines (such as updated sepsis bundles or hand hygiene protocols) are rapidly disseminated and adopted. In our study, some risk factors like “private insurance” raising POS odds is not intuitive—this might reflect subtle differences in clinical practice patterns. Ensuring all staff adhere to standardized best practices for every patient, regardless of assumptions about their background or coverage, is critical.

Third, enhanced surveillance and research should continue to monitor pediatric adverse events and evaluate the effectiveness of interventions. Administrative databases, such as the HCUP KID, are invaluable for identifying national trends and high-level risk factors; however, prospective studies and quality improvement registries should complement them. Health systems can use real-time dashboards to track their PDI rates and compare against benchmarks. Further research could explore the causal pathways underlying our findings. For instance, why do Western hospitals have higher POS rates? Is it due to the case mix, or could it be differences in antibiotic stewardship or surgical techniques? Why did private insurance associate with higher sepsis risk—could it indicate differences in hospital coding, or perhaps that privately insured patients might undergo more elective surgeries (with potential complications)? Additionally, qualitative research (e.g., provider interviews, patient focus groups) could illuminate barriers to optimal postoperative sepsis care and generate ideas for intervention.

Risk stratification tools could be developed to predict which pediatric surgical patients are most likely to develop sepsis, using the identified factors (and others, such as laboratory values). A formal risk score applied perioperatively might enable pre-emptive actions for those at highest risk [17]. For example, a high-risk child might be triaged to a pediatric ICU post-surgery for closer monitoring and early goal-directed therapy if needed. Some institutions are already employing advanced analytics and machine learning on EHR data to flag early warning signs of sepsis; incorporating pediatric-specific risk factors from studies like ours can improve their predictive accuracy.

Finally, quality improvement initiatives should be rigorously tested and shared. If a specific hospital or region has achieved notably low POS rates, understanding their strategies (whether it is a strict bundle, a novel infection control technology, or organizational changes) could benefit others. Collaboration through pediatric healthcare networks and publications in forums allows successful interventions to be scaled up. Given that our study supports the use of PDIs as useful quality metrics [6,7], hospitals should continue to measure these indicators and strive to reduce them. For instance, targeted programs to reduce postoperative complications (POS) might include implementing pediatric-specific surgical checklists that emphasize infection control, optimizing perioperative antibiotic use (including appropriate selection and duration), and post-discharge follow-up to catch and treat evolving infections early. Each of these should be evaluated for effectiveness. Randomized trials or stepped-wedge implementation studies could test interventions such as prophylactic probiotics or immunomodulators in high-risk surgical patients to see if sepsis can be prevented.

### 4.2. Strengths and Limitations

This study has several notable strengths. First, it utilizes the 2019 HCUP KID, the largest publicly available all-payer pediatric inpatient dataset in the United States. This allows for a nationally representative analysis of POS across diverse hospital settings and patient populations. Second, the application of the AHRQ Pediatric Quality Indicator 10 (PDI 10) provides a standardized, validated measure of POS, enabling consistency with other published studies and facilitating benchmarking across institutions. Third, the use of multivariable logistic regression, which incorporates both patient- and hospital-level covariates, permits adjusted estimates that better reflect independent risk factors and contextual effects. Finally, this study builds upon prior work examining NBSI and PORF to contribute a comparative, serial evaluation of high-priority pediatric adverse events.

However, several limitations must also be acknowledged. The analysis relies on administrative data, which may be subject to miscoding, underreporting, or variability in documentation practices across institutions. The PDI 10 definition is dependent on ICD-10-CM/PCS codes. It does not distinguish between preventable and non-preventable cases of POS, nor does it capture clinical severity (e.g., organ dysfunction, infection source, or culture results). While the dataset is robust, it lacks granular clinical variables such as intraoperative factors, antimicrobial prophylaxis timing, or postoperative monitoring protocols, which may influence POS risk but are not captured in claims data. The use of the “major OR procedure” variable must also be acknowledged as a limitation, as it serves only as a proxy for surgical exposure and does not capture the specific nature, complexity, or risk level of individual procedures. Another limitation inherent to the use of the HCUP KID is that it is an administrative dataset that does not contain any patient identifiers. As a result, it is not possible to track repeated admissions or differentiate between index stays and readmissions for the same patient. Furthermore, transfers between hospitals cannot be accurately tracked or linked between facilities. Moreover, the cross-sectional design precludes causal inference, and the outcome reflects association rather than direct attribution. Lastly, while we adjusted for numerous confounders, residual confounding from unmeasured variables, such as immunologic status, surgical complexity scoring, or staffing patterns, may persist.

Despite these limitations, the findings offer valuable insights into the epidemiology of POS in pediatric surgical care and provide a strong foundation for future investigations using more clinically enriched datasets.

## 5. Conclusions

POS is a significant and potentially preventable threat to pediatric surgical patients. Our analysis of a national inpatient database reveals that the risk of POS is multifactorial, shaped by the environment of care (hospital size, teaching status, region, and ownership) and the circumstances of a patient’s hospitalization (type of service, major surgery performed, etc.). Demographic factors like race/ethnicity and insurance status contribute to disparities that warrant targeted attention, although age and sex by themselves do not appear to drive risk. Many of the determinants of POS identified in this study (such as hospital-related factors and the need for major surgery) highlight areas where system-level improvements and best practices can make a difference. By investing in robust infection control programs, equitable care delivery, and ongoing staff training, healthcare institutions can reduce the incidence of postoperative sepsis. Furthermore, applying risk stratification and focusing resources on the highest-risk patients, without neglecting baseline standards for all, will improve overall outcomes. In summary, reducing pediatric POS will require both diligent clinical care and strategic public health efforts. The insights from this nationally representative study provide a foundation for developing interventions and policies to ensure that children recover safely after surgery, regardless of their background or where they receive care.

## Figures and Tables

**Table 1:** Comprehensive overview of POS (PDI 10) and key hospital/patient characteristics in the 2019 KID.

Variable	Attribute	Frequency	Percent
**POS (PDI 10)**	Absent	3,987,774	87.35%
	Present	577,625	12.65%
**Hospital bed size**	Small	470,770	15.20%
	Medium	742,057	24.00%
	Large	1,876,456	60.70%
**Hospital location**	Rural	189,298	6.10%
	Urban non-teaching	356,963	11.60%
	Urban teaching	2,543,022	82.30%
**Hospital region**	Northeast	529,073	17.10%
	Midwest	696,645	22.60%
	South	1,183,705	38.30%
	West	679,860	22.00%
**Hospital ownership**	Public	365,784	11.80%
	Private, not-for-profit	2,382,758	77.10%
	Private, investor-owned	340,741	11.00%
**Gender**	Female	1,587,394	51.40%
	Male	1,500,745	48.60%
**Race**	White	1,407,652	45.60%
	Black	513,619	16.60%
	Hispanic	607,329	19.70%
	Asian/Pacific Islander	123,698	4.00%
	Native American	26,306	0.90%
	Other	188,042	6.10%
**Service line**	Maternal and Neonatal	1,716,825	55.60%
	Mental Health/Substance Use	209,939	6.80%
	Injury	97,434	3.20%
	Surgical	219,576	7.10%
	Medical	845,509	27.40%
**Payment source**	Medicare	10,554	0.30%
	Medicaid	1,567,452	50.70%
	Private insurance	1,270,547	41.10%
	Self-pay	131,918	4.30%
	No charge	3843	0.10%
	Other	100,660	3.30%
**Operation on record**	No operation on record	2,718,390	88.00%
	Major operation on record	370,893	12.00%
**Variable**	**Attributes**	**Mean**	**Std Dev**
**Age**	N = 4,571,036	5.76	7.82

Abbreviations: POS, postoperative sepsis; PDIs, Pediatric Quality Indicators; and Std Dev, standard deviation.

**Table 2:** PDI 10 postoperative sepsis events (PPPD10), logistic regression (multivariate analysis).

Variable		Estimate	SE	Wald Test	*p*-Value	Adjusted OR	Wald 95% Confidence Limits for IRR	
**Intercept**		−1.1956	0.00969	15,223.4432	<0.0001	0.303	-	-
**Age**		0.000133	0.000239	0.3095	0.5780	1.000	1.000	1.001
**Sex**	Female	0.00328	0.00305	1.1558	0.2823	1.003	0.997	1.009
	Male *							
**Race**	White *							
	Black	0.00668	0.00432	2.3928	0.1219	1.007	0.998	1.015
	Hispanic	−0.0318	0.00413	59.3682	<0.0001	0.969	0.961	0.977
	Asian/Pacific Islander	0.00636	0.00742	0.7340	0.3916	1.006	0.992	1.021
	Native American	0.0802	0.0141	32.4814	<0.0001	1.084	1.054	1.114
	Others	0.0573	0.00625	83.8824	<0.0001	1.059	1.046	1.072
**Service line**	Maternal and Neonatal	−0.3752	0.00380	9764.0955	<0.0001	0.687	0.682	0.692
	Mental Health/Substance Use	−0.2396	0.00686	1221.0395	<0.0001	0.787	0.776	0.798
	Injury	0.2318	0.00808	822.7288	<0.0001	1.261	1.241	1.281
	Surgical	0.2728	0.00889	942.0917	<0.0001	1.314	1.291	1.337
	Medical *							
**Payment source**	Medicare	0.0301	0.0257	1.3687	0.2420	1.031	0.980	1.084
	Medicaid	−0.0197	0.00734	7.1986	0.0073	0.981	0.967	0.995
	Private insurance	0.0423	0.00744	32.3544	<0.0001	1.043	1.028	1.059
	No charge	0.3047	0.0354	74.1019	<0.0001	1.356	1.265	1.454
	Other	0.0701	0.0105	44.5725	<0.0001	1.073	1.051	1.095
	Self-pay *							
**Operation on record**	Major operation on record	0.1631	0.00728	501.9870	<0.0001	1.177	1.160	1.194
**Hospital bed size**	Small	−0.3318	0.00404	6728.2117	<0.0001	0.718	0.712	0.723
	Medium	−0.2408	0.00364	4379.8857	<0.0001	0.786	0.780	0.792
	Large *							
**Hospital location**	Rural	−1.0109	0.00574	31,018.6116	<0.0001	0.364	0.360	0.368
	Urban non-teaching	−0.6532	0.00484	18,213.8946	<0.0001	0.520	0.515	0.525
	Urban teaching *							
**Hospital region**	Northeast	−0.1931	0.00481	1613.2327	<0.0001	0.824	0.817	0.832
	Midwest	−0.2549	0.00460	3076.2645	<0.0001	0.775	0.768	0.782
	South	−0.2210	0.00398	3077.9496	<0.0001	0.802	0.795	0.808
	West *							
**Hospital ownership**	Public	−0.0209	0.00592	12.4474	0.0004	0.979	0.968	0.991
	Private, not-for-profit	−0.0940	0.00472	396.9995	<0.0001	0.910	0.902	0.919
	Private, investor-owned *							

Asterisks * indicate reference category.

## Data Availability

The original data presented in the study are openly available in AHRQ’s HCUP KID at https://hcup-us.ahrq.gov/db/nation/kid/kiddbdocumentation.jsp (accessed on 29 October 2022).

## Abbreviations

The following abbreviations are used in this manuscript:

AHRQ	Agency for Healthcare Research and Quality
POS	Postoperative sepsis
CLABSI	Central-line-associated bloodstream infection
HCUP	Healthcare Cost and Utilization Project
KID	Kids’ Inpatient Database
PDI	Pediatric Quality Indicator
PDI 10	Pediatric Quality Indicator 10 (postoperative sepsis)
NBSI	Neonatal bloodstream infection
NQI 03	Neonatal Quality Indicator 03
PORF	Pediatric postoperative respiratory failure

## Appendix A. Classification of Major Operating Room Procedures (PCLASS_ORPROC)

### The Data Element PCLASS_ORPROC Indicates Whether a Major Operating Room Procedure Was Reported on the Discharge Record [9]

The presence of operating room procedures reported on a discharge record was determined using the Procedure Classes Refined for ICD-10-PCS. Each ICD-10-PCS procedure code on the record was classified into one of four procedure classes:

- Minor diagnostic—Non-operating room procedures that are diagnostic (e.g., BW2800Z CT scan of head using high osmolar contrast);
- Minor therapeutic—Non-operating room procedures that are therapeutic (e.g., 079030Z drainage of head lymph, percutaneous approach);
- Major diagnostic—All procedures considered valid operating room procedures by the Diagnosis-Related Group (DRG) grouper and that are performed for diagnostic reasons (e.g., 00B00ZX excision of brain, open approach, diagnostic);
- Major therapeutic—All procedures considered valid operating room procedures by the Diagnosis-Related Group (DRG) grouper and that are performed for therapeutic reasons (e.g., 021008W bypass coronary artery, one artery from aorta with zooplastic tissue, open approach).

If at least one major diagnostic or major therapeutic procedure was included on the record, then PCLASS_ORPROC was set to 1. More information on the Procedure Classes (https://hcup-us.ahrq.gov/toolssoftware/procedureicd10/procedure_icd10.jsp) (accessed on 29 October 2022) is available under Tools & Software on the HCUP-US website.

Prior to the data year 2020, the major operating room procedure indicators were stored in the data element I10_ORPROC.

**Table A1:** Major Operating Room value description of data element.

Variable	Description	Value	Value Description
PCLASS_ORPROC	Major operating room ICD-10-PCS procedure indicator	0	No major operating room procedure reported on discharge record
		1	Major operating room procedure reported on discharge record
